# Template-Assisted Iron Nanowire Formation at Different Electrolyte Temperatures

**DOI:** 10.3390/ma14154080

**Published:** 2021-07-22

**Authors:** Malgorzata Kac, Anna Mis, Beata Dubiel, Kazimierz Kowalski, Arkadiusz Zarzycki, Iwona Dobosz

**Affiliations:** 1 Institute of Nuclear Physics, Polish Academy of Sciences, PL-31342 Kraków, Poland; anna.mis@ifj.edu.pl (A.M.); arkadiusz.zarzycki@ifj.edu.pl (A.Z.); 2 AGH University of Science and Technology, al. A. Mickiewicza 30, 30-059 Kraków, Poland; beata.dubiel@agh.edu.pl (B.D.); kazimerz.kowalski@agh.edu.pl (K.K.); iwona.dobosz@agh.edu.pl (I.D.)

**Keywords:** Fe nanowires, template-assisted electrodeposition, magnetic properties, polycarbonate membranes

## Abstract

We studied the morphology, structure, and magnetic properties of Fe nanowires that were electrodeposited as a function of the electrolyte temperature. The nucleation mechanism followed instantaneous growth. At low temperatures, we observed an increase of the total charge reduced into the templates, thus suggesting a significant increase in the degree of pore filling. Scanning electron microscopy images revealed smooth nanowires without any characteristic features that would differentiate their morphology as a function of the electrolyte temperature. X-ray photoelectron spectroscopy studies indicated the presence of a polycarbonate coating that covered the nanowires and protected them against oxidation. The X-ray diffraction measurements showed peaks coming from the polycrystalline Fe bcc structure without any traces of the oxide phases. The crystallite size decreased with an increasing electrolyte temperature. The transmission electron microscopy measurements proved the fine-crystalline structure and revealed elongated crystallite shapes with a columnar arrangement along the nanowire. Mössbauer studies indicated a deviation in the magnetization vector from the normal direction, which agrees with the SQUID measurements. An increase in the electrolyte temperature caused a rise in the out of the membrane plane coercivity. The studies showed the oxidation resistance of the Fe nanowires deposited at elevated electrolyte temperatures.

## 1. Introduction

Materials of a size that is comparable to the electron mean free path, i.e., with limited dimensions, have attracted much attention from the technological, engineering, and scientific points of view in recent years. One-dimensional nanostructures such as nanowires, because of their remarkable size- and shape-dependent, magnetic, optical, and electrical properties, are especially attractive materials that could have a wide range of potential applications in different fields such as biomedicine [1,2,3], spintronics [4,5], environmental protection [6,7], and consumer electronics [8,9,10,11,12,13]. Because of their large aspect ratio, defined as the ratio between the nanowire length and the diameter (L/ɸ), nanowires exhibit magnetic anisotropy, which makes them better candidates for MRI (magnetic resonance imaging) contrast agents, electrochemical water splitting, hyperthermia, or targeted drug delivery [3,14,15,16,17,18,19,20] than their spherical nanoparticle counterparts. The nanowire matrix, which has a high surface-to-volume ratio, enables the development of corrosion, flow, acoustic, and pressure sensors that have a significant efficiency enhancement [21,22,23,24,25,26]. When nanowires are embedded in polycarbonate membranes, they can serve as flexible permanent magnets [26,27,28,29,30], but when they are released from the template, they may act as responsive magnetic sensors [31,32]. The arrays of non-interactive magnetic nanowires that have a perpendicular anisotropy are being studied for use as materials for high-density magnetic recording media of several dozen terabits per square inch in the near future [33,34,35]. The variety of parameters that influence nanowire morphology, structure, and magnetic properties makes them attractive materials for fundamental studies. Developments in understanding the elementary principles of nanowire growth mechanisms enable researchers to precisely control their properties, thus creating characteristics that are superior to those of planar structures [36].

There are many different methods for producing metallic nanowires, including a broad spectrum of lithography techniques [37,38], direct evaporation [39,40] or simple and low-cost electrochemical deposition [41,42]. This last technique is becoming an increasingly attractive method of nanostructure synthesis. Its versatility and suitability for large-scale production because of its well-defined shape and crystalline characteristics are greatly needed for nanowire applications. 

The template-assisted method combined with the electrochemical technique is a general approach for preparing the desired material into a matrix of cylindrical pores. The porous membranes enable the production nanowires with a monodispersed diameter and a readily controlled length during unidirectional growth. The most popular alumina templates, which have hexagonally arranged high-density porous structures, are characterized by a small distance between pores, which together with their regular ordering, results in a strong dipolar interaction between the nanowires [20,43,44,45,46,47,48]. The small interpore distance is also the main cause of difficulties in contacting a single wire for use in GMR spin-valve devices [5] or studies of their mechanical properties such as strength measurements. In contrast, the track-etched polycarbonate membranes, which have a low pore density, enable the creation of quasi-separated nanowires with low or neglected dipolar interaction [41,49] and easy top-contacted wires for electrical and strength measurements [5]. Moreover, polycarbonate membranes are available in a wide range of thicknesses, pore sizes, and densities as low as a single pore per membrane. Additionally, nanowires that are embedded in these membranes are promising materials for flexible electronic applications.

Because of their biocompatibility, neutrality, and easy intracellular digestion [50], unlike Ni- and Co-containing nanostructures, iron nanowires are attractive materials for chemical and biomedical applications [17]. The crucial problem during nanowire deposition is the pore filling efficiency, which can be controlled by chemical agents, the electrodeposition rate, or other electrodeposition parameters such as pH, temperature, electrolyte composition, values of cathodic potential, or current [51,52,53]. In the presence of air, iron nanowires naturally develop an oxide layer on their surface, which makes them biocompatible and biodegradable, but that results in limitations in their sensor-like applications; thus, preventing the uncontrollable oxidation of nanowires is another challenge in the production of Fe nanowires [26]. 

Here, we report on the fabrication of iron nanowires via template-assisted electrodeposition in polycarbonate membranes. The growth mechanism of the nanowires and their morphology, structure, magnetic properties, and pore-filling ratio were studied as a function of the electrolyte temperature. Furthermore, the Fe nanowire oxidation resistance was also analyzed to determine the chemical stability of Fe nanowires deposited at an elevated electrolyte temperature.

## 2. Materials and Methods

**Figure 1:**
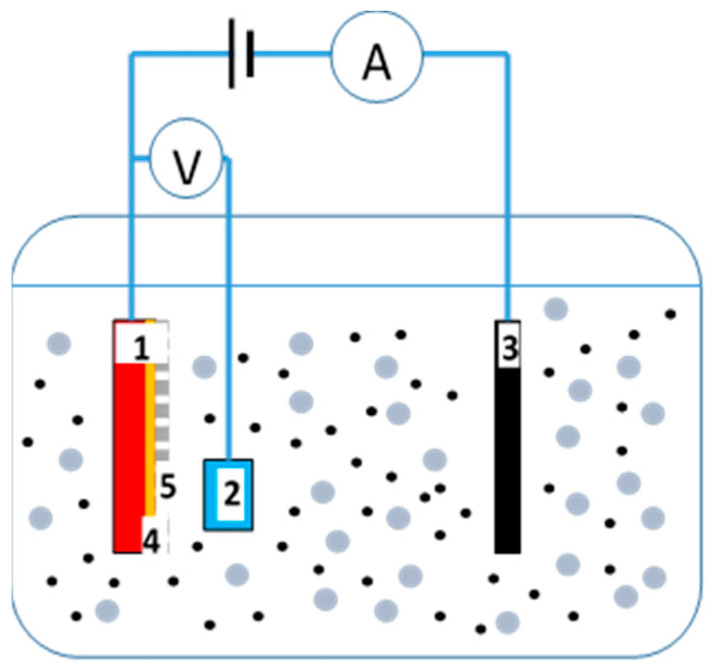
The electrochemical cell with a three-electrode system: 1—working electrode (cathode), 2—reference electrode, 3—counter electrode, 4—copper contact layer, 5—membrane.

Iron nanowires were deposited in polycarbonate membranes that were purchased from the Sterlitech Corporation (Kent, Ohio, USA). The electrodeposition process was performed in an electrochemical cell (Figure 1) in a three-electrode system with a platinum sheet and Ag/AgCl, which served as the counter (3) and reference electrodes (2), respectively. The working electrode (1) was prepared before the nanowires were grown by sputtering a thin copper contact layer (4) on one side of the membranes (5). The electrodeposition process was controlled by the AUTOLAB PGSTAT302N potentiostat (Metrohm Autolab B.V., Utrecht, The Netherlands) operating in the potentiostatic mode at the cathodic potential of −1.1 V vs. Ag/AgCl. The membranes that were used in the experiment showed inhomogeneous pore distribution [41] with a pore diameter of 100 nm, a pore density equal to 4 × 10^8^ pors/cm^2^, a porosity of 3.1%, and a nominal membrane thickness of 6 µm. The chemicals, which were of analytical grade at concentrations of 0.4 M FeSO_4_ × 7H_2_O, 0.7M H_3_BO_3,_ and 1 g/L C_6_H_8_O_6_ and deionized water (resistivity >18 M·Ω·cm) from the Millipore system, were used to prepare the electrolytes. The pH was adjusted to 2.7 using 2M NaOH. The electrodeposition process was performed at electrolyte temperatures that ranged from 15 °C to 40 °C. 

The pore-filling and morphology of the nanowires were observed using a scanning electron microscope (SEM) (Tescan Vega 3, Tescan Orsay Holding, a.s., Brno, Chech Republic) before and after a membrane dissolution in dichloromethane. The structure of the nanowires embedded in the polycarbonate membrane was investigated by means of X-ray diffraction (XRD) using a X’Pert MRD Pro diffractometer (Malvern Panalytical Ltd, Malvern, UK) with Cu Kα radiation operating at 40 kV and 30 mA in θ–2θ geometry. Additionally, the microstructure of the nanowires was examined with a transmission electron microscope (TEM) using the FEI Tecnai G2 20 X-TWIN electron microscope (FEI, Hillsboro, Oregon, USA) that was equipped with a LaB_6_ emission source. The indexing of the electron diffraction patterns was performed with the use of JEMS software (version 4.4131U2016) by P. Stadelmann (JEMS-SWISS, Jongny, Switzerland).

The magnetic properties of as-prepared nanowires were measured at room temperature using a superconducting quantum interference device (SQUID) magnetometer (MPMS, Quantum Design, San Diego, California, USA) by applying an external field of up to 4 T in the plane and out of the plane of the membrane. The diamagnetic signal of the sample holder and the polycarbonate membrane was subtracted from the hysteresis loops. In addition, the oxidation state of the Fe nanowires was analyzed using X-ray photoelectron spectroscopy (XPS) and Mössbauer spectroscopy. The XPS measurements were performed in a vacuum system workshop (with a residual pressure below 5 × 10^−8^ mbar during analyses), operating at Mg Kα radiation of energy 1253.6 eV. A concentric hemispherical electron analyzer worked in a fixed analyzer transmission mode with a constant pass energy of electrons set at 22.5 eV. The binding energy scale was calibrated by fixing the position of the dominant C1s peak of the adventitious carbon to 284.6 eV. The Mössbauer studies were conducted in transmission geometry using a 100 mCi ^57^Co(Rh) source and a He–10% CH_4_ gas flow counter. The measurements were taken at room temperature, and the direction of the γ-ray propagation was perpendicular to the sample surface. The Mössbauer spectra were fitted as the sum of the Lorentzian sites using Recoil software, ver. 1.03a (D. G. Rancourt, Ottawa, ON, Canada). 

## 3. Results and Discussion

The experimental conditions of the electrochemical process, such as the current density, potential, electrolyte composition, and temperature, had significant effects on the morphology and properties of electrodeposited materials. They are, therefore, crucial points in order to understand and control these properties [54]. 

### 3.1. Electrochemical Analysis

**Figure 2:**
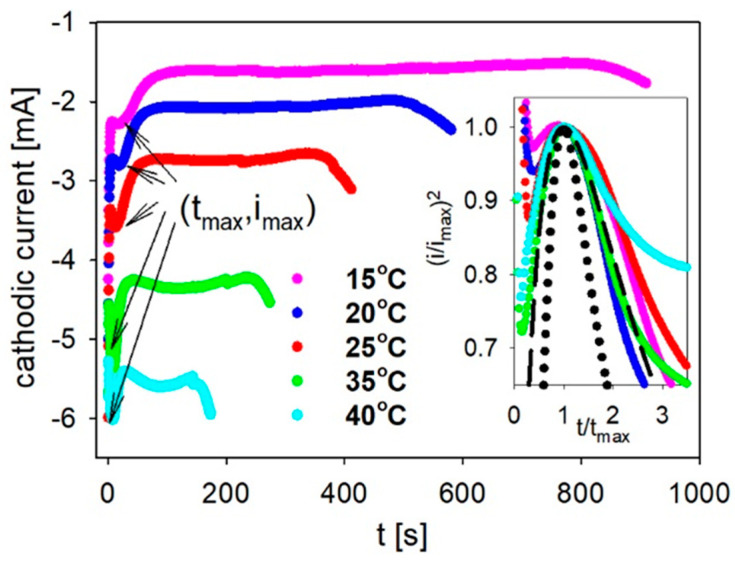
The cathodic current density versus the time measured during the deposition of the Fe nanowires that were prepared at different electrolyte temperatures. The inset shows the dimensionless peaks (derived from the current transients) with theoretical curves for an instantaneous (dashed curve) or progressive nucleation (dotted curve).

**Figure 3:**
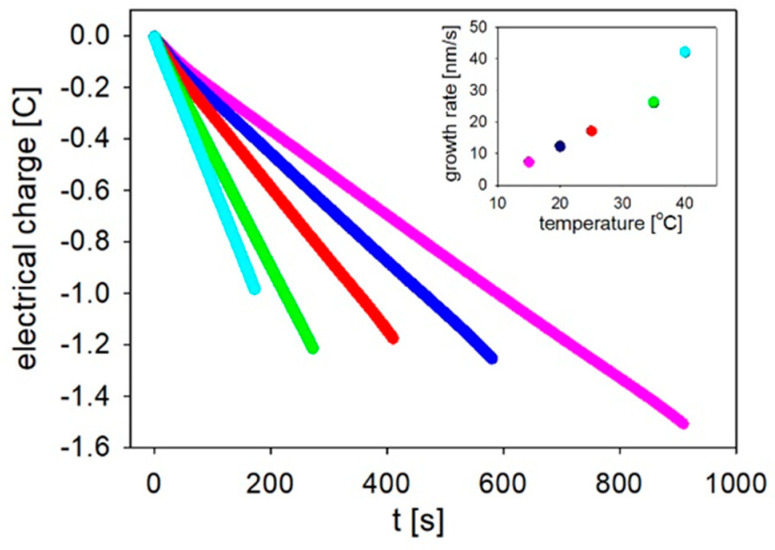
The electrical charge that was reduced into the porous membranes versus the time measured during the deposition of the Fe nanowires that were prepared at different electrolyte temperatures. The inset shows the growth rate as a function of the electrolyte temperature.

We followed the deposition process by monitoring the cathodic current (Figure 2) and electrical charge transients (Figure 3) that were registered at different electrolyte temperatures as a function of time. In the current transient, which was measured during the pore-filling process, four different zones could be distinguished. When the potential was applied, the high current values (with a sharp decrease) at the very beginning were linked to a large number of ions in the close vicinity of the cathode surface before double layer formation. After that, there was a sharp increase in the current (first zone) associated with the charging of the electrical double layer. A reduction of the Fe^2+^ located directly at the cathode surface then occurred, which was connected to the decrease of the current. A concentration gradient that resulted in a flux of ions toward the cathode is responsible for the formation of the diffusion layer. In the second zone, in which the current nearly remained constant, electrodeposition into the pores took place. When the nanowires reached the membrane surface, there was an increase in the current (third zone), and the caps started to grow on the polycarbonate [41,52,55]. The further continuation of the electrodeposition process could result in the expansion of the caps on the membrane surface and the creation of the continuous layer (fourth zone), which would be indicated by a nearly constant current value. This last zone could not be seen because the deposition process was stopped when an increase in the current provided information regarding the filling of the pores and the beginning of the formation of the over-deposited caps (see Section 3.2.)

A decrease in the electrolyte temperature significantly extended the electrodeposition time and caused a reduction of the cathodic current (in the absolute value). At higher temperatures, the conductivity of the solution increased due to an increasing ion activity, which resulted in a lower required overpotential, which, at the same value of the applied potential, caused an increase of the cathodic current. A higher overvoltage can favor the side reaction as hydrogen evolution [55]. 

The electrolyte temperature affects the diffusion of metal ions and, together with migration, which is controlled by the overpotential, results in a variation of the electrodeposition rate [42]. The growth rate that was calculated as a function of the electrolyte temperature is shown in the inset in Figure 3.

At the same time, the electrical charge, which was reduced into the pores, was also registered (Figure 3). The total charge measured during the processes conducted at the medium temperatures (20–35 °C) had nearly the same values, while at the extreme temperatures, it differed significantly (0.52 C). A high value of the reduced charge during a slow electrodeposition process might be caused by an increased pore filling degree and a less porous morphology of nanowires due to a lower hydrogen evolution [51]. Moreover, by lowering the electrolyte temperature, in addition to an enhancement of the pore-filling degree, a large-scale uniformity in length can simultaneously be achieved [52]. On the other hand, a high temperature promotes hydrogen evolution, which inhibits the metal ion reduction and results in an inhomogeneous deposition [56,57,58,59]. A decrease of the pore-filling degree and a strong hydrogen bubble evolution that blocked the pores when the electrolyte temperature was increased were also reported by Azevedo et al. [51].

During electrodeposition, two processes occur: nucleation, which is followed by the formation of new grains, and the growth of existing nuclei. Because the nucleation process is dependent on the deposition temperature [52], it would be worth analyzing. 

As was mentioned above, the sharp peaks (indicated by arrows in Figure 2) that correspond to double-layer charging were followed by a decrease in the current due to the diffusion-limited process. At this electrodeposition step, a linear diffusion inside the pores is achieved [60]. Moreover, the mass transport within the nanopores is limited by the diffusion along the whole channel length [52,61]. A diffusion-controlled process is also observed during the spherical diffusion in the fourth stage of electrodeposition, in which the overdeposition occurs. This conclusion is based on the observation that at the fourth step (creation of the continuous layer on the membrane surface), the current merely doubled, although the surface area increased several dozen times [41,62].

These peaks (Figure 2), which were converted to the dimensionless plot that is shown in the inset as the relationship between (*i*/*i_max_*)^2^ and (*t*/*t_max_*), enabled the dominant nucleation mechanism to be identified. The values of the i_max_ and t_max_ are the maximal cathodic current and the time at which it was reached, respectively. The nucleation process is characterized by the frequency of nuclei appearance and the corresponding rate at which active sites are depleted. When the nuclei are formed at a very early stage, the nucleation is classified as instantaneous. In contrast, when the nuclei are formed continuously during crystallite growth, the nucleation mechanism is classified as progressive [53,63,64]. In the case of instantaneous nucleation, the relationship between *(i/i_max_)^2^* and *(t/t_max_)* follows the equation [65,66]:

(1) $${\left(i/i_{max}\right)}^{2}=1.9542{\left(t/t_{max}\right)}^{-1}\left(1-\exp\left[-1.2564\;(t/t_{max}\right)\right]{)}^{2}$$

and is graphically presented as a dashed curve in the inset in Figure 1. The progressive nucleation according to Equation (2) is shown as a dotted line [65,66].

(2) $${\left(i/i_{max}\right)}^{2}=1.2254{\left(t/t_{max}\right)}^{-1}\left(1-\exp[-2.3367{\left(t/t_{max}\right)}^{2}\right]{)}^{2}$$

It is evident that the curves that were obtained for the nanowires deposited at 20 °C and 35 °C follow the response that is predicted for instantaneous nucleation (the nucleation is fast and occurs on a relatively small number of active sites that are exhausted at an early stage of the process [65]) precisely, although other experimental data also agreed quite well. This type of nucleation process was expected because of the limitations that were connected with the small open area of the polycarbonate membranes and the long nanochannels, which might result in a small number of active sites. From the kinetic analysis, it was also possible to obtain information concerning the diffusion coefficient (D) according to Equation (3) [65,66]:

(3) $$i_{max}{}^{2}t_{max}=0.1629{\left(zFc\right)}^{2}D$$

where: *z*—the number of exchanged electrons, *F*—the Faraday constant, *c*—the ion concentration. 

**Figure 4:**
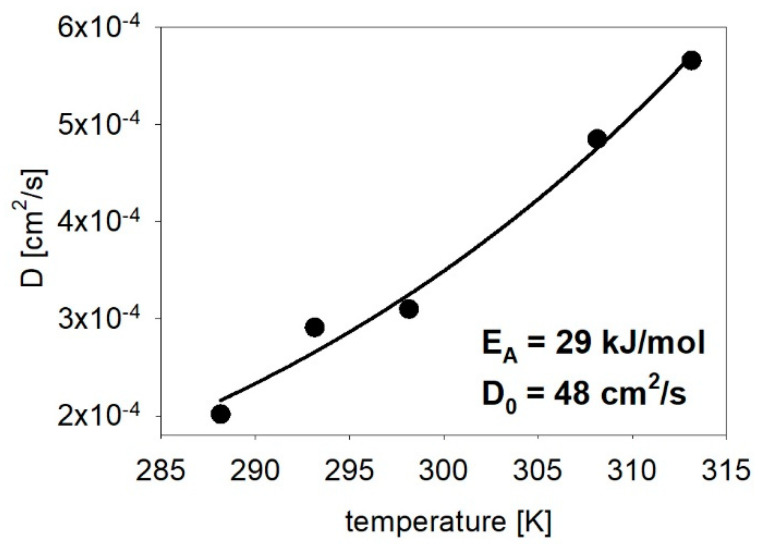
The temperature dependence of the calculated diffusion coefficient with the activation energy and pre-exponential factor values that were estimated from the Arrhenius equation.

The values of the diffusion coefficient calculated based on Equation (3) were presented as a function of the electrolyte temperature in Figure 4 together with the line taken from the Arrhenius equation, which describes the temperature dependence of the diffusion coefficient (D = D_0_ exp(−E_A_/RT). This equation enables the determination of the activation energy and pre-exponential factor (diffusion coefficient at an infinite temperature). The assumed surface in the current density value corresponded to the open area of the membrane and did not take into account the difference in the pore-filling degree.

An increase in the electrolyte temperature resulted in a rise in the diffusion coefficient in accordance with the Arrhenius equation (Figure 4). The activation energy is close to the value for iron oxidation process in a sulfuric acid solution [67,68] or ferrous diffusion in chloride [69]. The obtained value is also close to the activation energy of silver diffusion in ferric sulfate [70] and ferric diffusion in various sulfate solutions [71]. This value is typical of a reaction whose kinetics are diffusion-controlled processes [70]. A relatively small value of the activation energy increases the diffusion coefficient because less thermal energy is required to overcome the smaller activation energy barrier [72]. 

The diffusion coefficient values were higher than the value that was calculated for the Fe^2+^ ions in water at 25 °C, which was equal to 7.19 × 10^−6^ cm^2^/s [73], and the diffusion coefficient that was measured for ferrous sulfate using a different gel type that varied between 1.9 × 10^−7^ and 6.1 × 10^−6^ cm^2^/s [74]. The diffusion coefficient values were one order larger than the values that were estimated using the Einstein–Smoluchowski equation [75] or that were calculated in chloride at a high temperature [69]. On the other hand, the values were very close to the diffusion coefficient that was obtained by Pruna et al. for zinc oxide nanowires that had been electrodeposited in polycarbonate membranes [76] and by Manzano [60] in a diluted electrolyte in alumina membranes. These high values could be connected to hydrogen evolution, which showed a larger diffusion coefficient than Fe ions [75]. The decreased diffusion coefficient and the ion concentration gradient due to the lower deposition temperature effectively reduces ion diffusion rate, thereby favoring uniform growth [52]. This is in accordance with the large value of electrical charge that was reduced into the nanochannels at the lowest electrolyte temperature. Moreover, not only was the diffusion coefficient decreased, but the thickness of the diffusion layer also elongated as the temperature was decreased [52]. 

### 3.2. Structure and Morphology

**Figure 5:**
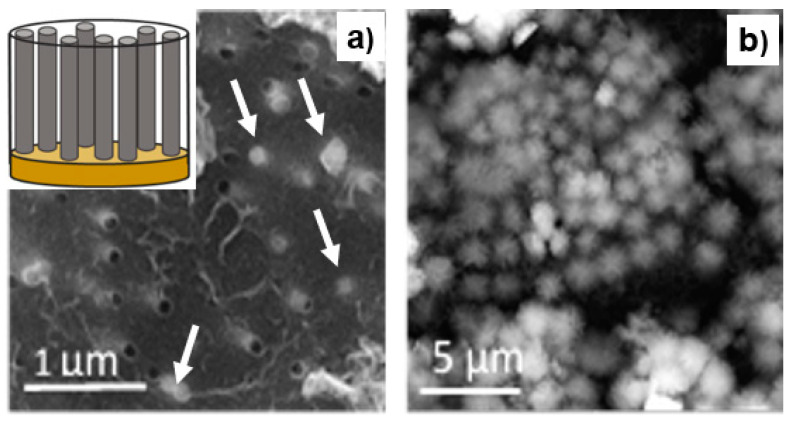
Scanning electron microscopy images of the membrane surface with a Fe nanowire deposition that stopped when the nanowires reached the membrane surface (**a**) and then continued up to the formation of the over-deposited caps (**b**). The arrows indicate the nanowires which reached the membrane surface. The scheme shows nanowires in the membrane, standing on the copper contact layer.

As mentioned above, the electrodeposition processes were stopped when the nanowires reached the membrane surface (Figure 5a). The arrows indicate the nanowires that reached the membrane surface. The others are close to the surface and are observed as a white background around the pores. The specially prepared, additional sample with over-deposited caps did not reveal any features that could identify the nanowire structure [15,77] due to the presence of an oxide layer [3] that completely covered the caps (Figure 5b).

**Figure 6:**
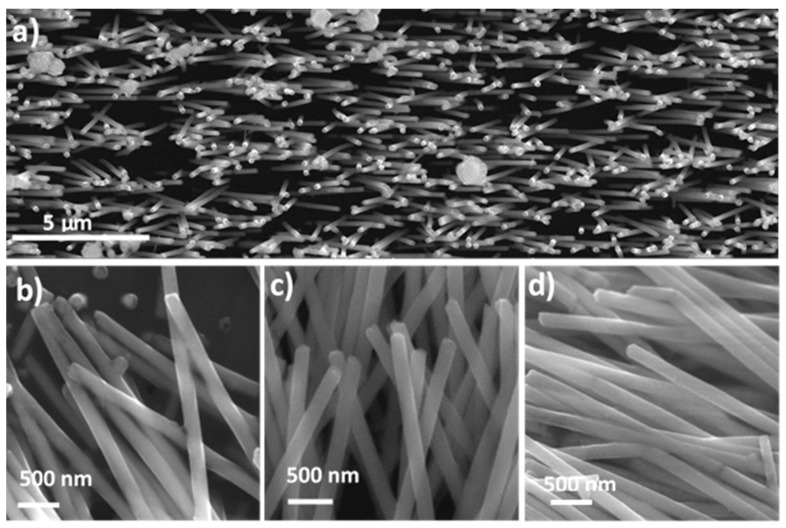
Scanning electron microscopy images of the Fe nanowires observed after membrane dissolution and measured for the nanowires that were deposited at the different electrolyte temperatures: 15 °C (**b**), 25 °C (**a**,**c**), and 40 °C (**d**).

The morphology of the nanowires that was observed after membrane dissolution is shown on the SEM images in Figure 6. The nanowires did not reveal any significant changes as a function of the electrolyte temperature. The vertically oriented nanowires created a uniform nanowire matrix. In all of the cases, densely packed nanostructures were shown, which confirmed the complete filling of the membranes independent of the electrodeposition rate or hydrogen evolution. The nanowires that were observed at a higher magnification had a continuous structure with a smooth, at this scale, lateral surface with a narrow diameter distribution without any trace of porosity.

**Figure 7:**
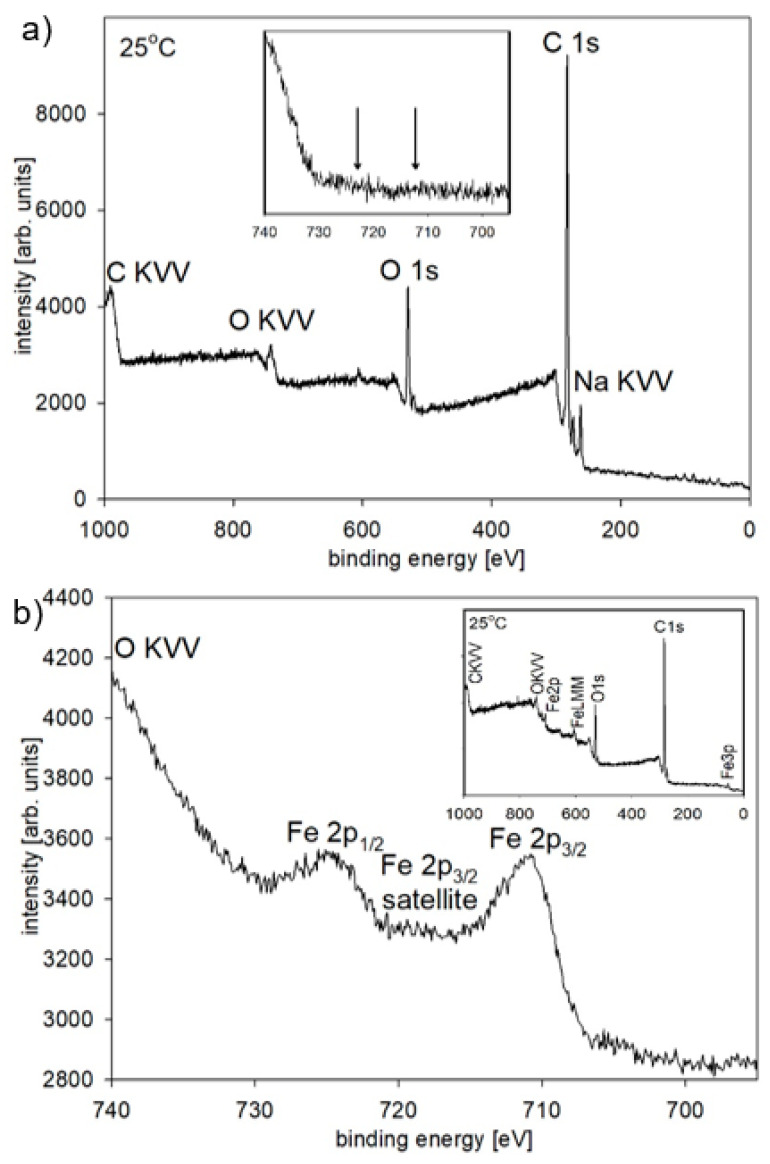
The XPS spectra of the Fe nanowires were measured after membrane dissolution (**a**), and on the over-deposited caps formed on the membrane surface—(**b**). The insets show the detail (**a**) and survey scans (**b**). The arrows indicate the positions of the Fe2p_1/2_ and Fe 2p_3/2_ peaks.

The morphology of the nanowires that were observed on the SEM images after membrane dissolution did not show any trace of oxide formation, but the diameter of the nanowires, which was estimated based on the SEM patterns, was a few tens of nanometers larger than the pore size. This suggests a presence of a coating that had formed on the nanowire surface. To identify its chemical composition, X-ray photoelectron spectroscopy measurements were taken (Figure 7a).

The XPS spectrum showed peaks that were not characteristic for either Fe oxide or metallic Fe (inset Figure 7a), which, taking into account the surface sensitivity of this method, proves the presence of a thin iron-free layer on the nanowire surface. The registered elements, especially the high-intensity carbon peak (which at this amount could not be assigned to a surface impurity), indicate a layer of polycarbonate that had covered the nanowires during the dissolving process. The appearance of the small intensity sodium peak, which is a characteristic of membrane contamination that is introduced during the etching of the latent ion tracks, was an additional confirmation of the presence of a polycarbonate coating. The significant background under the peaks was caused by inelastically scattered electrons. A rough estimation of the thickness of the polycarbonate coating, which was performed based on the SEM image and the XPS sensitivity, indicated a layer of 20–30 nm. This coating protected the Fe nanowires against oxidation in contrast to the over-deposited caps that had been extended on the membrane surface that, when kept at ambient atmosphere, were immediately covered by an oxide layer that can be seen on the SEM image (Figure 5b). The XPS analysis that was conducted on this surface revealed a high-spin multiplet-split Fe2p spectrum (Figure 7b). The peaks at binding energies of 710.9 eV and 724.6 eV corresponded to the Fe2p_3/2_ and Fe2p_1/2_ photoelectron states, which, together with the shake-up satellite of Fe2p_3/2_, confirmed the presence of iron in oxidation state +3 (Fe_2_O_3_) [78,79]. The absence of metallic Fe suggests that the thickness of the oxide layer exceeded 10 nm.

The presence of this thin coating, according to our knowledge, was not reported by other groups. Obviously, it can be observed only in nanowires produced in polycarbonate membranes and is connected to the membrane dissolution procedure.

**Figure 8:**
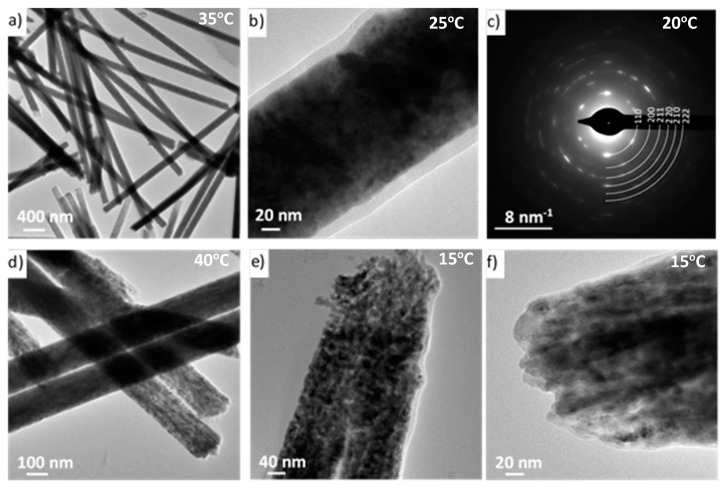
TEM images of Fe nanowires in bright-field mode with diffraction pattern (**c**) indexed with the lattice parameters of bcc Fe structure (**a**) 35 °C, (**b**) 25 °C, (**c**) 20 °C, (**d**) 40 °C, (**e**,**f**) 15 °C.

The microstructure of the Fe nanowires was investigated using transmission electron microscopy (TEM). Figure 8a shows an overview of the TEM image of the nanowires with a uniform diameter of about 140 nm. On the lateral surface of a nanowire, a thin amorphous layer (identified as a polycarbonate by XPS) with a 10–15 nm thickness is visible (Figure 8b). The bright-field TEM image revealed a fine-crystalline structure with crystallite sizes from a few to 30 nm (Figure 8d–f) and shapes that varied from elongated to equiaxial. The fine crystallites grew in a columnar direction along the nanowire axis (Figure 8d,f). The diffraction patterns consisted of semicontinuous rings and elongated spots, which confirmed the polycrystalline bcc Fe structure of the nanowires (Figure 8c). The samples did not have any significant differences in their microstructure as a function of the electrolyte temperature.

**Figure 9:**
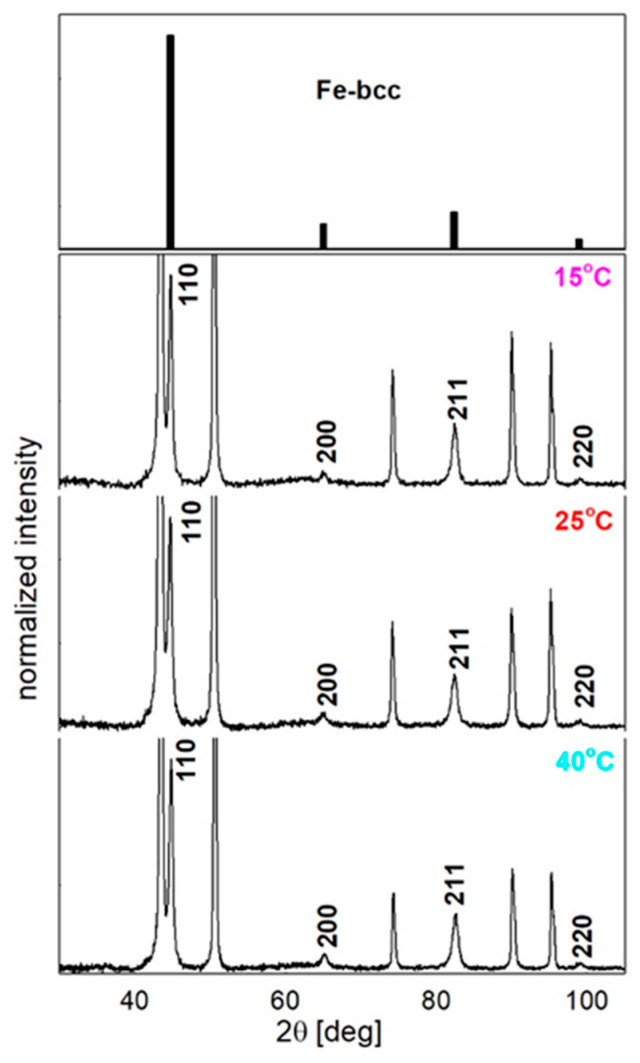
X-ray diffraction patterns measured for the Fe nanowires embedded in the polycarbonate membranes. The indexed peaks corresponded to the bcc Fe powder sample (reference code: 03-065-4899 NIST Database), while the peaks, which are not described, correspond to the copper contact layer.

The structure of the nanowires was also analyzed based on X-ray diffraction. In Figure 9, the XRD spectra of the nanowires that were deposited at the lowest, medium, and highest electrolyte temperatures together with the reference sample are presented. The most intensive narrow, non-indexed peaks resulted from the copper contact layer that was sputtered on one side of the membrane. The indexed peaks correspond to the polycrystalline Fe in the bcc structure and appeared in all of the spectra with only a slight difference in their intensity and widths. The relative peak intensities that correlate with a powder sample (reference code 03-065-4899 NIST Database) suggest a slight texture that prefers the growth of (211) planes. Moreover, the calculations of the texture coefficient, which was determined using the Harris formula [80], indicated that this preferential orientation attenuates with increasing temperature, and for the highest temperature, the crystallite growth became almost isotropic.

**Figure 10:**
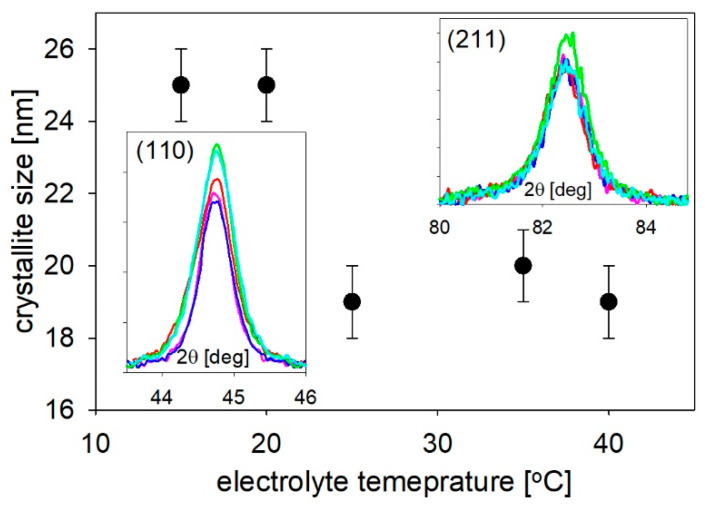
The mean value of crystallite size was calculated according to Scherer’s formula based on the (110) and (211) peaks. The color of the lines corresponds to the description presented in Figure 2 and Figure 3.

A slight broadening of the peaks with an increasing electrolyte temperature was observed for the most intensive (110) and (211) reflections, which suggests a decrease of the crystallite size at higher temperatures. The results of the calculation that were performed based using Scherrer’s equation are shown in Figure 10. The mean values of the crystallite sizes, estimated with an error of ±1 nm, were equal to approximately 25 nm at the lower electrolyte temperatures and about 20 nm at the higher temperatures (along the nanowire axis). This reveals a clear tendency to reduce the crystallite size with an increasing electrolyte temperature. In the case of the potentiostatic mode of the electrodeposition, the increase in the electrolyte temperatures is associated with an increase in the current density. It is the main reason for the rise in deposition rate, which causes the reduction in crystalline size. Additionally, higher temperatures promote hydrogen evolution, which could also block crystalline growth. The mean crystallite size is consistent with the values that were estimated based on the TEM images.

A similar observation was made by Azevedo et al. [51], who reported a decrease in grain size at a higher electrolyte temperature and explained it as a local increase of current density. Based on XRD results, Shin et al. [52] reported that the electrolyte temperature did not cause any significant crystallinity changes; however, the diffraction pattern (TEM studies) indicated a rise in crystallite size at higher temperatures. An increase in the crystallite size with an increasing electrolyte temperature was also observed by Saeki et al. [42]. It was elucidated as the rise in the surface diffusion that favors the growth of the preexisting nuclei that were established during the first stages of the electrochemical process.

### 3.3. Mössbauer Measurements

**Figure 11:**
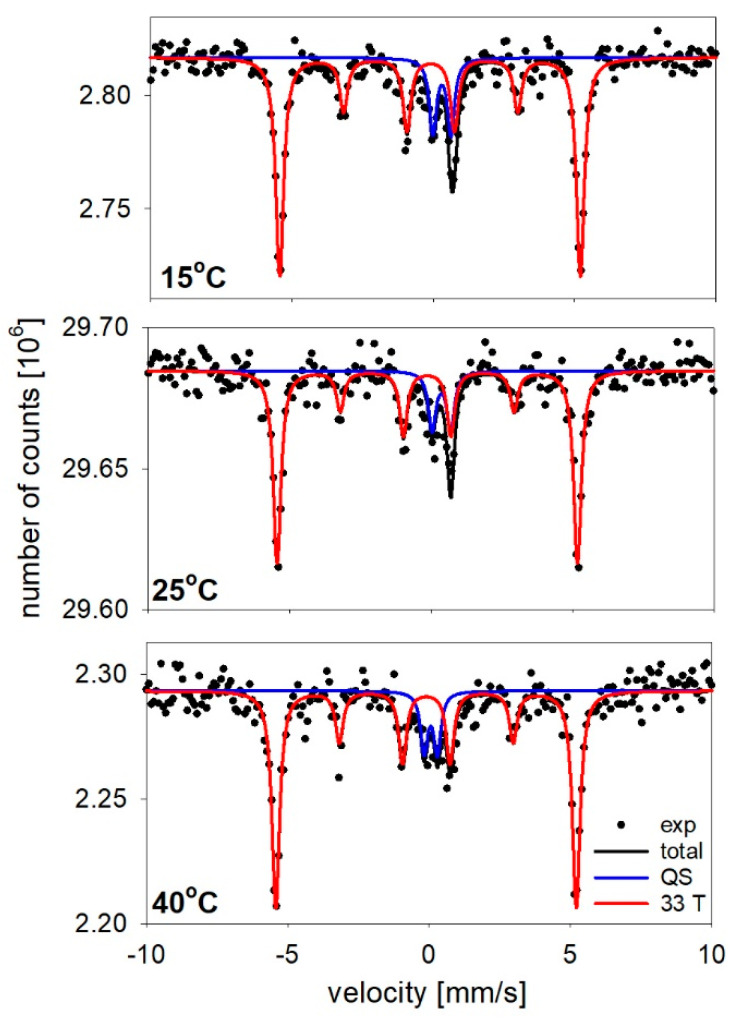
Mössbauer spectra measured at room temperature for the nanowires embedded in the polycarbonate membranes shown along with fitted components: doublet (QS) and sextet (33T).

The phase composition of the nanowires was studied using Mössbauer spectroscopy measurements. This technique enables iron as well as iron-based compounds (especially iron oxide) to be detected. The Mössbauer spectra, which were measured at room temperature for the nanowires embedded in the polycarbonate membranes, are presented in Figure 11. All of the spectra were fitted with two components that were described by hyperfine parameters, which are listed in Table 1. 

The dominant component of the spectra was a sextet with a relative contribution of about 80%. This component was characterized by a hyperfine magnetic field that was equal to approximately 33 T, an isomer shift (IS) close to −0.10 mm/s, and nearly 0 vales of quadrupole splitting (QS), which together are typical for bulk-like Fe atoms [81,82]. The contribution to this component came from the Fe nucleus surrounded by Fe atoms that were arranged in a regular structure. The 0 value of quadrupole splitting especially suggests rather relatively large grains with a well-ordered cubic structure. The second- and third-line intensity ratio (A2/A3), which is shown in Table 1, provides information regarding the relationship between the γ-ray propagation and the magnetization vector orientation (θ). The estimated value of θ varied from 30° to 33°, which indicated the deviation of the magnetization vector from the normal direction.

The second component of the spectrum was a doublet with a relative contribution of about 20%. This component was defined by an isomer shift and quadrupole splitting. The doublet indicated Fe atoms in a paramagnetic or superparamagnetic state. While the sextets were assigned to Fe atoms inside the nanowires, the doublets could be associated with the Fe atoms at the edges or in the voids, in which there were a limited number of Fe atoms in a ferromagnetic state in the close neighborhood of the nucleus.

Because of the difficulties in measuring the Fe nanowires connected with the very small amount of Mössbauer isotope, which was caused by the low abundance of ^57^Fe in the natural iron (2%) and a low membrane porosity (3%), we did not observe a close correlation between hyperfine parameters and the electrolyte temperature. However, these studies, together with the XRD measurements, indicated that the nanowires were composed of iron without any iron oxide contamination independent of the electrolyte temperature.

### 3.4. Magnetic Properties

**Figure 12:**
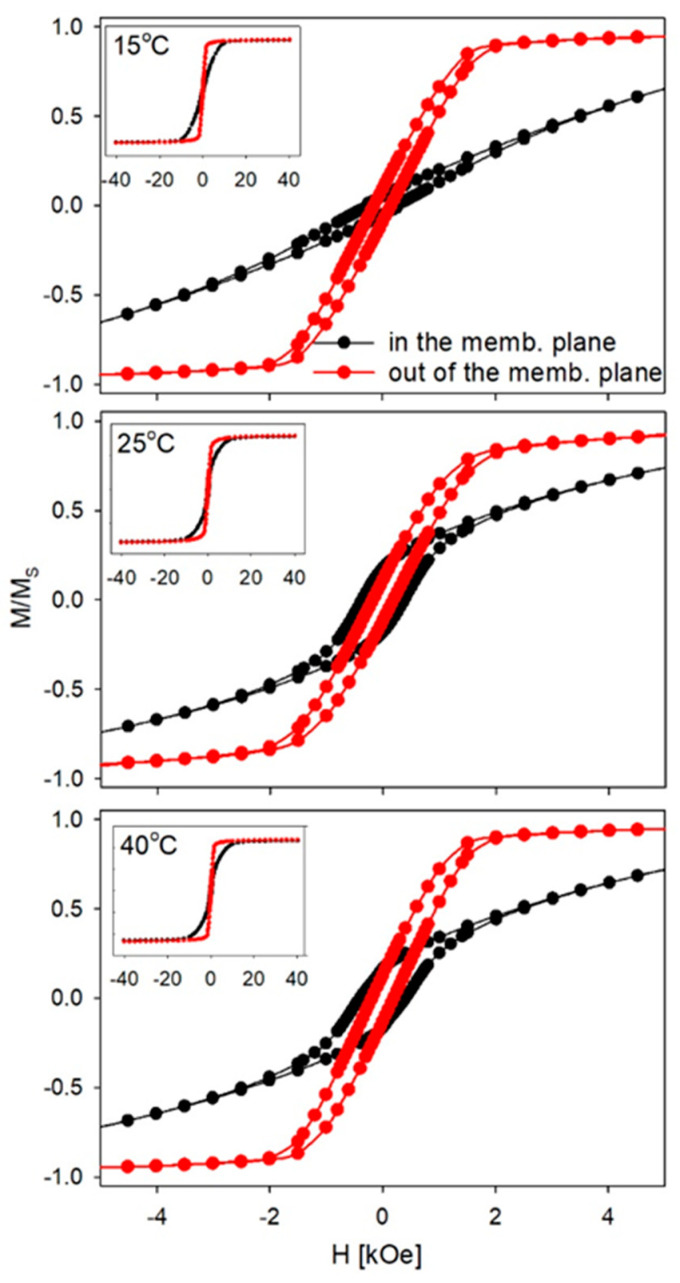
Hysteresis loops measured at room temperature in the membrane plane and out of the membrane plane. The magnetization (M) value was normalized to the saturation magnetization (M_S_).

The magnetic measurements of the Fe nanowires embedded in the polycarbonate were performed at room temperature with the magnetic field applied out of the membrane plane and in the membrane plane. The hysteresis loops of the Fe nanowires deposited at the lowest, medium, and highest electrolyte temperatures are presented in Figure 12. 

All of the measured samples exhibited magnetic anisotropy that had an easy axis of magnetization close to the nanowire axis. Generally, the effective anisotropy, in this case, was a result of the magnetocrystalline and shape anisotropy [41,83]. Because of the polycrystalline structure of the Fe nanowires and the slight crystallographic texture, which did not correlate to magnetic properties, we can conclude that the shape anisotropy is a dominant factor that determines the observed preferred orientation of the magnetization vector [84]. The tilting of the hysteresis loops, which was measured in the easy direction, suggests a deviation of the magnetization vector from the normal direction or a significant contribution of the dipolar interaction in the magnetic behavior of the Fe nanowire matrix [44]. However, the non-regular distribution of the nanowires and the relatively large average nanowire distance that was connected to the low membrane porosity (the dipole interaction decrease with a power of 3 of the nanowire distance) indicated a relatively minor impact of dipolar interaction. Taking the results of the Mössbauer measurements into account, we can conclude that the observed tilting was mainly related to the deviation of the nanowire axis from the normal direction that was caused by the imperfect orientation of nanochannels, which resulted in a tilted magnetic easy axis. Such a deviation could be the reason for the open hysteresis curves that were observed in both directions. The only exception of the curve that was measured in the membrane plane for the nanowires that were deposited at the lowest electrolyte temperature was that it indicated a really hard direction of magnetization in which the magnetization rotated coherently towards the field direction [61].

The magnetic measurements with the field applied out of the membrane plane indicated low coercivity values. Coercivity is significantly affected by the aspect ratio, which for values above 28, results in a significant reduction of the coercivity [61] and drops twice in the range of 30–120 [41]. Thus, the aspect ratio of L/Φ= 60 was the main factor that was responsible for a low value of the coercive field. 

**Figure 13:**
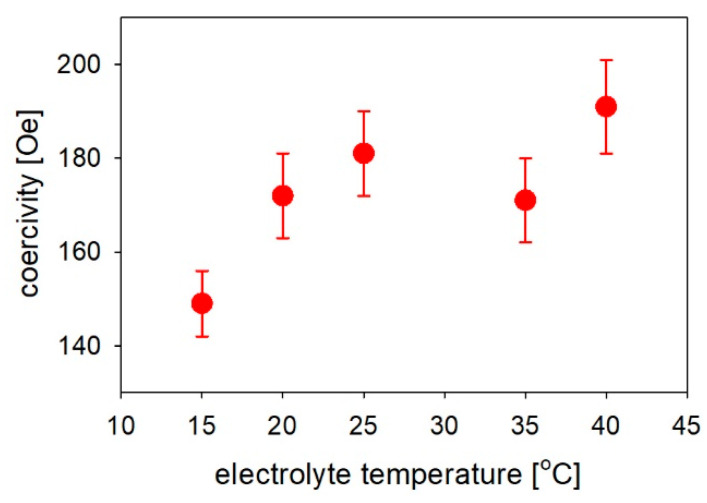
Coercivity values versus electrolyte temperature measured for the Fe nanowires with the magnetic field applied out of the membrane plane.

The coercivity values (Figure 13) increased with electrolyte temperature. The elevated electrolyte temperature was associated with higher hydrogen evolution, which was responsible for a larger number of defects. The more defected nanowire structure at elevated temperatures may be a reason for the larger coercivity because of the pinning of the domain walls caused by defects. Similarly, Schlörb et al. observed an increase in the coercivity for irregularly shaped nanowires compared to the low values for the smooth nanowires prepared under optimized conditions [61]. 

## 4. Conclusions

We studied Fe nanowires deposited at various electrolyte temperatures. A cathodic current analysis indicated an instantaneous nucleation model. The calculated values of the diffusion coefficients followed the Arrhenius plot. The electrical charge reduced into the channels increased at the lowest electrolyte temperature, which suggested a higher pore-filling degree. The Fe nanowires grew in a polycrystalline bcc structure with a slight texture along the [211] direction and the crystallite size decreasing at higher temperatures. All of the nanowires exhibited a magnetic anisotropy with an easy axis that deviated from the normal direction and a small value of coercivity that increased as a function of the electrolyte temperature. The nanowires were spontaneously covered by a polycarbonate coating during membrane dissolution that protected them against oxidation, which opens up new application possibilities.

## Figures and Tables

**Table 1:** The hyperfine parameters of the spectra that were measured for the nanowires deposited at different electrolyte temperatures.

Electrolyte Temperature	Subspectra	IS [mm/s]	QS [mm/s]	B_hf_ [T]	A_2_/A_3_	θ	Relative Contribution
15 °C	doublet	0.31	0.63	–	–	–	16
	sextet	−0.10	−0.01	32.97	0.7	33	84
20 °C	doublet	0.26	0.65	–	–	–	23
	sextet	−0.10	0.00	32.98	0.6	30	77
25 °C	doublet	0.37	0.65	–	–	–	16
	sextet	−0.13	0.00	32.94	0.6	30	84
35 °C	doublet	0.06	0.46	–	–	–	13
	sextet	−0.11	−0.01	32.93	0.7	33	87
40 °C	doublet	0.06	0.46	–	–	–	14
	sextet	−0.10	0.00	32.96	0.70	33	86

## Data Availability

The data is available within the article and can be requested from the corresponding author.
